# Dual-Gate Organic Thin-Film Transistor and Multiplexer Chips for the Next Generation of Flexible EG-ISFET Sensor Chips

**DOI:** 10.3390/s23146577

**Published:** 2023-07-21

**Authors:** Ashkan Rezaee, Jordi Carrabina

**Affiliations:** Department of Microelectronic and System, Autonomous University of Barcelona, 08193 Barcelona, Spain; jordi.carrabina@uab.cat

**Keywords:** OTFT, EG-ISFET, multiplexer, dual-gate OTFT, electrostatic discharge

## Abstract

Ion-sensitive field-effect transistors (ISFETs) are used as elementary devices to build many types of chemical sensors and biosensors. Organic thin-film transistor (OTFT) ISFETs use either small molecules or polymers as semiconductors together with an additive manufacturing process of much lower cost than standard silicon sensors and have the additional advantage of being environmentally friendly. OTFT ISFETs’ drawbacks include limited sensitivity and higher variability. In this paper, we propose a novel design technique for integrating extended-gate OTFT ISFETs (OTFT EG-ISFETs) together with dual-gate OTFT multiplexers (MUXs) made in the same process. The achieved results show that our OTFT ISFET sensors are of the state of the art of the literature. Our microsystem architecture enables switching between the different ISFETs implemented in the chip. In the case of sensors with the same gain, we have a fault-tolerant architecture since we are able to replace the faulty sensor with a fault-free one on the chip. For a chip including sensors with different gains, an external processor can select the sensor with the required sensitivity.

## 1. Introduction

Thin-film transistor (TFT) technology has advanced significantly and has been widely employed in many fields since the creation of the first TFT in 1962 [1,2,3]. The fast advancement of liquid crystal display (LCD) technology in the 1970s presented new opportunities for TFTs. TFTs were best suited to fulfill the LCD industry’s urgent need for a semiconductor switch device that could drive a big-area active matrix (AM) on glass [4]. As a result of this urge, amorphous silicon (a-Si)-based TFT devices were introduced by Le Comber et al., to be used in AM LCD (AMLCD) in 1979 [5]. A year later, hydrogenated a-Si (a-Si:H) met the demand of AMLCD since its mobility improved significantly [6].

TFT technology has advanced continuously along with the growth of flat panel display (FPD) technology, which employed the TFT as its fundamental component. This lead to a technology evolution similar to silicon, with a main focus on size rather than integration density.

The mobility of an a-Si:H TFT is insufficient for the FPD devices, which need a higher TFT mobility to optimize driving performance. In 1980, Depp et al., illustrated that poly-crystalline silicon (p-Si) TFT devices, with a typical carrier mobility of 10^2^ cm^2^ V^−1^ s^−1^, are an excellent alternative for meeting high-resolution and huge-current driving demands [7]. Even though TFT mobility reached 50 cm^2^ V^−1^ s^−1^, the process temperature of more than 300 °C severely limited its practical application. As a result, many procedures for producing low-temperature polysilicon were tested (LTPS) [8]. Serikawa et al., were the first to use laser irradiation to lower the annealing temperature of polysilicon in 1989 [9]. However, the commercial promotion of TFTs was constrained because it needed to be annealed at 300 °C to obtain solid results. By lowering the LTPS process temperature to less than 150 °C, LTPS TFT arrays could be manufactured on the same glass substrate as amorphous Si TFT arrays [10]. When compared to an a-Si:H TFT, the carrier mobility of an LTPS TFT can be tens to hundreds of times greater. The advantages of LTPS LCDs over conventional a-Si:H LCDs include quick response times, excellent resolution, and high aperture ratio. With the advancement of internet of things (IOT) technology in recent years, TFT arrays have also been used in the low-cost IC industry. Biggs et al., published a 32-bit ARM (a reduced instruction-set computing architecture) microprocessor constructed with metal-oxide TFT technology on a flexible substrate in 2021, paving the way for the development of low-cost, fully flexible, smart integrated systems [11].

Many different novel materials have been utilized in attempts to make TFTs in addition to the traditional silicon-based technologies. Organic semiconductors (OSCs) are the one among them that have been the subject of the most research.

Flexible electronics need transistors that work well under a specific range of mechanical deformation, and OSCs seem to be one of the best options for these semiconductors. 

The first organic thin-film transistor (OTFT), which used polyacetylene as the semiconductor, was described by Ebisawa et al. in 1983 [12]. Drury et al., developed flexible polyimide substrates for all-polymer integrated circuits in 1998 [13]. The OTFT device offers numerous advantages, including low cost, structural flexibility, and big-area fabrication based on a solution technique, but it is insufficient in performance uniformity and device stability [14,15,16]. As a result, the OTFT has the potential to be employed in a wide variety of applications, including electrophoretic displays, radio frequency identification (RFID), and biomedical sensors [17,18,19,20,21]. Furthermore, OTFTs may be used in the driving array of mini-LED backlight sources. 

Massive efforts in material chemistry and processing techniques have been made in recent decades to improve the device mobility of OTFTs [22,23,24,25,26,27]. In many envisioned power-constrained electronic systems, reducing leakage current is equally or even more critical [28]. The quality and thickness of the Organic Gate Insulator (OGI) layer determine how much leakage occurs through it, and patterning the OSC layer can efficiently control parasitic leakage out of the channel region [29,30,31]. The primary obstacle to be overcome is the intrinsic channel leakage current, which is contributed by small carrier injection from the drain contact [32]. The carrier mobility (µ) of OTFTs has now surpassed 2 cm^2^ V^−1^s^−1^, above that of amorphous silicon transistors (1 cm^2^ V^−1^s^−1^) [33,34,35,36]. However, considering the need of high-resolution displays, it is still lower than inorganic semiconductor materials like LTPS TFTs and oxide semiconductors like IGZO TFTs [37,38,39]. As a result, effectively boosting output current and improving reliability are critical concerns for OTFT commercialization.

Using a common p-type device as an illustrative example, minority carrier electrons might be injected from the drain electrode into the lowest unoccupied molecular orbital (LUMO) states of the OSC with the application of a moderately positive gate voltage for the OFF-state or deep subthreshold regime. For narrow energy bandgap (Eg) OSCs, such as donor-acceptor (D-A) structure molecules, which have been extensively researched for high mobility because of their strong intermolecular contacts permitting efficient intermolecular charge transport, the minority carrier injection is more severe [40,41]. Performance instability was also brought on by the trapped electrons in the OSC layer or at the interfaces [42,43]. In order to reduce the negative consequences of minority carrier injection, two different types of techniques have been developed (electrons in p-type OTFTs) [44,45,46,47,48]. One method is to add molecular additives to the channel as specific charge-carrier electron traps, which prohibit the injected electrons from assisting in conduction [45,46]. However, it is challenging to manage this approach in processes for complete electron avoidance without interfering with the transit of holes in the ON-state [49]. The other technique is to limit electron injection by increasing the height of the electron injection barrier by contact doping or adding a broad interfacial layer at the contact interface [44,47,48]. The relatively poor charge-transfer doping achieved in OSCs, unlike that obtained with inorganic semiconductors, is not able to completely eliminate electron injection, and the dopants in the form of small-molecule counterions may migrate throughout the OSC layer, leading to device instability [50,51].

In this paper, for OTFTs, we propose to follow the same evolution that led from standard MOS transistors to build silicon MOS ISFETs using standard fabrication processes [52]. As was the case for silicon CMOS transistors, some changes must be made in the process technology to implement microsystems including sensors and circuitry. In our case, since we plan to use an extended gate, the required modification is the capability to change the threshold voltage. This is obtained from a dual-gate technology.

## 2. EG-ISFET Sensors

Microfluidics is a burgeoning area of study that deals with the microscale treatment of fluids; it is most often distinguished by objects that have critical dimensions less than 1 mm. Researchers can use a variety of physical laws that scale well at this scale, including quick diffusion [53], laminar flows [54], Dean flow [55], rapid heat transport [56], and taking advantage of the high surface area to volume ratio [57]. Microfluidics is used in a wide range of industries, including analytical chemistry [56], molecular biology [58], energy production [59], cell separations [55], and energy generation.

A large portion of the work on the subject of microfluidics has been carried out utilizing soft lithography, which Whitesides [60] first developed in 1998. Soft lithography techniques, particularly for polydimethylsiloxane (PDMS), have been extensively studied [61,62]. The need for cleanroom manufacturing, while well-developed by the microelectromechanical systems (MEMS) community, is still expensive and time-consuming, and this was one of the key challenges of soft lithography in the beginning. Recently, this has been somewhat mitigated by low-cost mold-making techniques, as discussed by Faustino [63]. In addition to soft lithography, other fabrication techniques for submillimeter channels have been developed by microfluidic engineers for several reasons, including lower costs, quicker turnaround times, less expensive materials and tools, and improved functionality.

One way to measure pH levels of microfluidics systems is using ion-sensitive field-effect transistors (ISFETs). ISFETs are a type of sensor that can detect changes in ion concentrations, including hydrogen ions (H^+^), which are responsible for pH. The ISFET consists of a thin film of metal oxide that acts as the sensing element and is placed near the gate of a field-effect transistor (FET). The FET modulates its threshold voltage in response to changes in the concentration of ions in the film, allowing for the detection of pH levels [64,65,66].

When monitoring electrochemical reactions, ISFETs have many benefits. ISFETs can be mass produced and further integrated into other sensing systems because they are based on the common complementary metal-oxide-semiconductor technique [64,65,66,67,68].

ISFETs can be implemented with OTFTs by connecting the top-gate electrode to the media where the pH measurement must be performed. In this way, the drain to source current will depend on the electrical charge related to the pH. Furthermore, pH electrode and gate electrode can be separated (while connected with a good conductor) to produce the extended-gate electrode. This allows for an easier integration of the electrode with the measurement environment and increases the level of reuse of the ISFET sensors, which are more expensive than electrodes and can trap chemical materials that degrade their behavior.

Ion-sensitive field-effect transistors with extended gates are known as EG-ISFETs. They are a particular kind of sensor that can determine the number of ions present in a solution by observing changes in the surface potential of a sensing oxide layer [69]. By adopting an extended conductive layer to separate the sensing oxide layer from the gate oxide of a thin-film transistor (TFT), the gate oxide is shielded from the electrolyte solution and the sensor’s stability and robustness are increased. Many applications, including biosensors, environmental monitoring, and food quality management, are possible with EG-ISFET sensors [70].

The simultaneous detection of multiple analytes in a single sample can be obtained by using several electrodes with different sensitive materials acting as a microelectrode array (MEA) on several EG-ISFETs. The multiplexing capabilities of EG-ISFETs makes them an effective tool for monitoring pH levels in cells as well as other ionic species.

The EG-ISFET has been used in a variety of applications, including the measurement of pH levels in cells, tissues, and biofluids [70,71,72,73]. It has been used in cancer cell research, where changes in pH levels can indicate the presence of cancerous cells [73]. It has also been used in stem cell research, where pH levels are used to monitor stem cell differentiation into specific cell types [74,75]. In addition to its use in medical applications, the EG-ISFET has also been used in environmental monitoring, where it can be used to measure pH levels in water and soil [76,77]. The EG-ISFET has also been used in food safety applications, where it can be used to detect the presence of harmful bacteria in food products [77].

## 3. Dual-Gate OTFT Technology 

Modern IC fabrication in CMOS technology nodes requires a lot of power and has a significant environmental impact [78]. SmartKem offers a more energy-efficient and eco-friendly process that involves organic materials and fewer fabrication steps than CMOS, which reduces cost and greenhouse emissions. 

The reduction of the complete fabrication process temperature from 180 °C to 80 °C has recently been demonstrated [79]. This ecofriendly fabrication process will: (1) require overall lower energy use in manufacturing (since no PECVD is required); (2) use a wider choice of plastics with improved properties concerning transparency, biodegradability (<12 months), bio-derived (e.g., cellulose), and low cost; and (3) be able to be integrated with other processes without destroying their devices (e.g., an OTFT backplane could be processed on top of the OLED device), which will also provide the potential for R2R manufacturing. This is one of the reasons to select the SmartKem OTFT low-temperature process to implement our integrated microsystems. 

Their spin-coated OTFT devices have mobilities of 2.5 cm^2^ V^−1^s^−1^ in a short channel (down to a channel length of 2.5 microns), low variability (<10%), and the option to turn on a voltage of +2 V to +4 V (on single-gate transistors). The process also enables digital design on OFETs (organic field-effect transistors) with non-complimentary logic and 3.3 V power supplies (or above). 

SmartKem devices can be fabricated on rigid (usually glass) or flexible (usually PEN) surfaces. OTFT devices on flexible substrates show good performance with bending [80] that allows for adapting their sensors and circuits to different environments (wearables, industrial, etc.). A less than 18% positive V_T_ shift in OTFT devices is observed under different bending conditions. This degradation comes from two mechanisms: the contribution of oxygen for a positive V_T_ shift and the contribution of mechanical tensile bending stress for a negative one. The main difference in degradation is the change in the molecular distance due to different bending conditions (tensile/compressive).

One method to lower electron injection by raising the height of the electron injection barrier is the dual-gate structure. The electrons can be repelled from the OSC layer, and the leakage current can be reduced by applying a negative voltage to the back gate [81,82,83]. 

SmartKem also introduced dual-gate OTFTs processing steps on their single-gate OTFT process, similar than other organic processes that implement digital circuitry [84,85]. In our case, these transistors play a crucial role in ensuring that the analog multiplexers effectively block the current from the unselected ISFET sensors [86,87].

**Figure 1:**
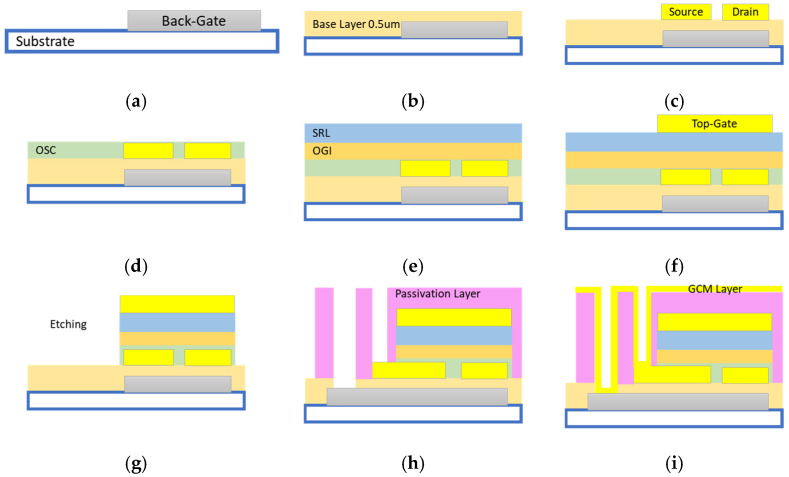
Different steps of SmartKem fabrication process: (**a**) Fabrication of first layer (Mo/Al/Mo back-gate metal) by sputtering, photolithography, and wet-etching (thickness: 11 nm/70 nm/60 nm). (**b**) Spin-coating and UV/thermal curing of base layer. (**c**) Sputtering, photolithography and wet-etching of second metal layer. (**d**) Spin-coating and baking of SAM and OSC layers (thickness: 30 nm OSC). (**e**) Spin-coating and baking of OGI layer, spin-coating and UV/thermal curing of SRL layer (thickness: 150 nm OGI and 400 nm SRL). (**f**) Sputtering, photolithography and wet-etching of third metal layer (thickness: 50 nm). (**g**) Spin-coating and UV/thermal curing of passivation layer (thickness: 2 um). (**h**) Patterning of passivation layer using photoresist and dry-etch transfer. (**i**) Sputtering, photolithography and wet-etching of fifth layer.

The dual-gate structure consists of five metal masks, as shown in Figure 1. The structure is provided by SmartKem Co., (Manchester, UK). In this strategy, the first mask is used to sputter and shape Mo/Al/Mo back-gate metal by photolithography and wet etching with the thicknesses of 11 nm/70 nm/60 nm (Figure 1a). The back-gate layer was then spin-coated with the base layer, which was then UV-cured (Figure 1b). This layer has a thickness of about 0.5 um. 

The second mask is devoted to gold deposition for the source and drain metals, is roughly 50 nm thick, and is photolithographically patterned (Figure 1c). Their single gate-OTFT process starts directly in step (c), involving the deposition and patterning of the source and drain metals.

Gate layer fabrication involves several stages. Self-assembled monolayers (SAM) and organic semi-conductors (OSC) with a 30 nm thickness are spin-coated and baked as the initial stage (Figure 1d). 

Next, the Organic Gate Insulator (OGI); dielectric sputter-resistant layer (SRL), which is an acrylate-based dielectric; spin-coating; and UV curing were applied (Figure 1e). The thicknesses of the OGI and SRL were 150 nm and 400 nm, respectively. Then, mask 3 was used, followed by a photolithographic pattern (Figure 1f). All additional OGI, SAM, SPL, and OSC were eliminated using mask 3 (Figure 1g). This layer was around 50 nm thick. The remaining region was then spin-coated with the passivation layer at a height of 2000 nm (Figure 1h). With the use of mask 4, the passivation layer copies the design using photoresist patterns. Finally, mask 5, also known as gate-contact metal, was used for patterning sputtered gold, and was wet-etched in a technique identical to that used with the previous metal layers (Figure 1i).

Electrostatic discharge (ESD), which can result in a very high current passing through the device or microchip in a very short amount of time and cause catastrophic irreparable damage, is one of the most common risks to the dependability of electronic components. In everyday life, electrostatic discharge (ESD) is a common occurrence. When two different-charged objects are near one another, the electric field either causes the insulating medium between them to break down and create a conductive path, neutralizing the charge transfer; alternatively, the different-charged objects directly contact one another, neutralizing the charge transfer. When a conductive path is created, ESD happens [88]. 

Silicon-based ISFETs suffer from ESD (on the sensitive-gate electrode), so silicon substrates allow for building protection circuits for their input-output pins (using diodes to connect the external inputs to the voltage supply sources). The substrates used for TFTs and OTFTs, especially those facing flexible systems, are usually dielectrics and do not offer the possibility to implement these circuits based on diodes. 

From the circuit point of view, one technique to gain fault-tolerance against ESD is to add redundancy. The ISFET destroyed by ESD can be substituted by another one in the same chip without the need to change the chip itself—just by correctly reconfiguring it. The low cost of OTFTs processes allow for integrating several devices in the same chip/die at a reduced cost increase.

For our study, we selected a substrate die size to fit one standard silicon chip package to demonstrate our concept while minimizing human contact when handling dies. A QFN64 package with a 9 mm × 9 mm area dimension and 64 pins defines the design die and allows for shielding it from ESD and light effects on OTFTs. Further flexible implementations will allow for these protections on encapsulations and substrates. 

### 3.1. Corbino-Shape Transistors

Circular-shaped transistors, also known as Corbino transistors, are less frequently employed in circuit designs; instead, this gate shape is more frequently used in the display field to open or close a light-emitting diode (LED). The channel in this transistor architecture is shaped like a ring. The Corbino disk, first reported by M. Corbino in 1911, is a disk with inner and outer concentric ring contacts [89]. It has been primarily used in magneto-resistance measurements [90] and has more recently been adopted for organic thin-film transistor (TFT) architectures [91,92].

An annular-shaped electrode was initially used in 1996 in hydrogenated amorphous silicon (a-Si:H) TFTs to give a lower gate-to-source capacitance and a lower photocurrent level in active-matrix liquid crystal displays (AM-LCDs) [93]. Ring-shaped and circular electrodes were utilized in a pseudometal-oxide semiconductor field-effect transistor in 1999 to describe the electrical properties of silicon-on-oxide wafers by device geometrical features [94].

For a given transistor width, the Corbino TFT’s circular channel design reduces the drain capacitance and resistance. By decreasing the impacts of parasitic capacitance and boosting switching speed and frequency response, this improves the device’s performance [95,96]. Furthermore, its circular symmetry produces a less variable behavior of OTFT organic semiconductors.

The Corbino TFT is appropriate for sensors and other high-speed signal processing applications. In order to prevent the direct current (DC) component of the input signal from having an impact on the output signal, the suggested sensor makes use of the Corbino TFT. The sensor’s operational point is set to the desired value by supplying a bias voltage to the Corbino TFT’s gate terminal [95]. As a result, the input signal may be measured by the sensor more precisely and steadily. Because of its distinctive circular form and low drain capacitance, the Corbino TFT is able to detect signals regardless of the input signal’s DC component. It also has a high switching speed. One example of such an application is a large-area active-matrix organic light-emitting diode (AMOLED) display pixel [97].

### 3.2. Interdigitated Transistors

The inverted/staggered and co-planar OTFT structures are the most widely used devices when looking for large transistor width [14]. Interdigitated electrodes are often used to compensate for the low OSC conductivity in OTFTs. The source and drain electrodes have a comb-like shape and their teeth are alternately interlocked with each other. This increases the channel width per unit of area and allows higher current density [98]. Maximizing the transistor width per unit of area allows us to place more transistors on the same substrate. However, this geometry still has some limitations in terms of performance, as it depends on the careful alignment of OSC crystals along the Drain and Source (D/S) electrodes during OSC deposition. This involves using a nitrogen flow, changing the temperature, and adjusting the solvent type and ratio [99,100,101].

## 4. Microsystems Chip Architecture

Our microsystems architecture merges several EG-ISFET sensors implemented with dual-gate OTFTs with analogue OTFT multiplexers in the same chip, to select, through a digital control, the sensor that will be used for the measurements. The main goal of this integration is to enhance the reusability of chips by selecting among several ISFET sensors without the need to change the chip. 

**Figure 2:**
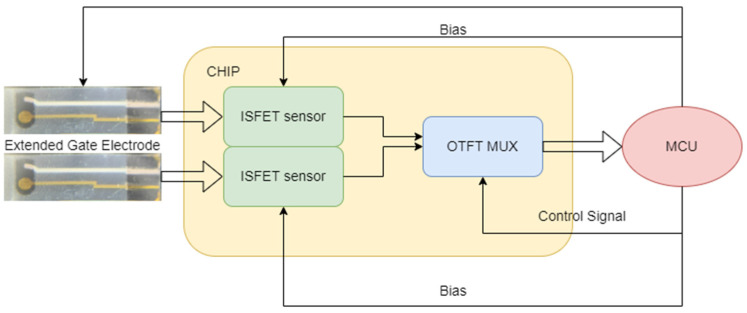
System architecture showcasing the integration of a pair of ISFETs and one MUX (one chip section) and its connection to external components (external electrodes and MCU).

The system architecture is illustrated in Figure 2, in which our chip will be connected to: (i) the extended gate and external electrode modules (left side); and (ii) a Microcontroller Unit (MCU) unit that will manage the control and bias of the chip (and external electrode) and will receive the analog signal from the ISFETs (routed by the multiplexers).

## 5. Multiplexing Sensor Signal

Analogue multiplexers are electronic devices that select one input signal from many and route it to a single output. They are widely used in a wide range of applications, including data acquisition systems, signal processing, and communication systems [102,103,104,105,106,107]. Analogue multiplexers can be built with a variety of technologies, including bipolar junction transistors (BJTs), field-effect transistors (FETs), metal oxide semiconductors (MOS) and OTFTs. Because of their distinct properties, OTFTs are a particularly appealing solution for multiplexer applications.

**Figure 3:**
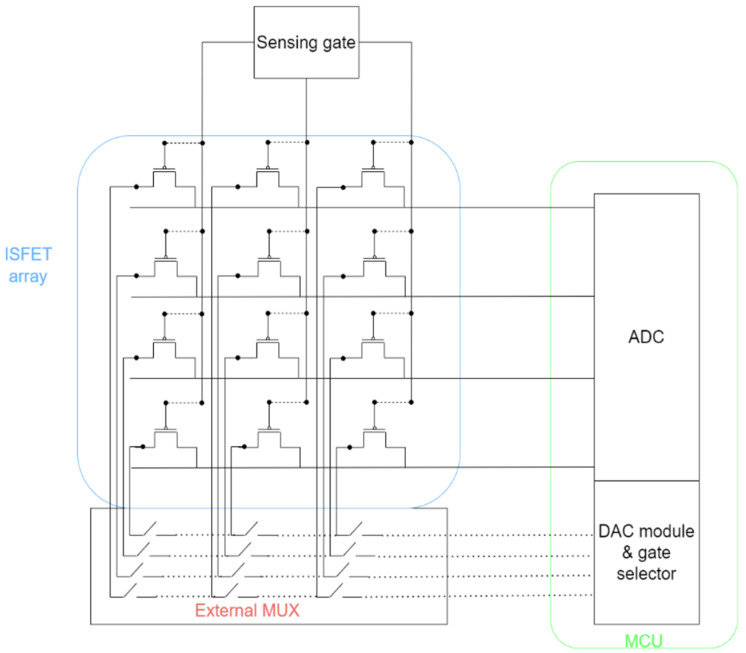
System-level architecture with focus on module interconnections and components.

A common approach for utilizing ISFET arrays is to employ MUXs and connect them to a microcontroller unit (MCU), as demonstrated in Figure 3 [102]. A series of select lines controls the matrix, which is used to pick the chosen input signal. The selected signal is then routed to the output after passing through a buffer amplifier.

One of the most difficult tasks in constructing an analogue multiplexer employing OTFTs is minimizing crosstalk between neighboring input signals [108,109]. The current running through one channel impacts the voltage at the input of an adjacent channel due to the low impedance between closer electrical nodes. This is referred to as crosstalk. This can result in a loss in signal quality and a decrease in the multiplexer’s overall performance. 

When compared to standard silicon-based transistors, the usage of OTFTs in multiplexer circuits allows for low-noise operation, good signal quality, and improved performance [110]. To provide best performance, the design of an analogue multiplexer based on OTFTs must consider crosstalk between neighboring input signals. 

With the continuous growth of OTFT technology and the development of new approaches for avoiding crosstalk, we may expect to see an increase in the number of analogue multiplexer applications that use OTFTs in the future [111,112].

## 6. Circuit Design

**Figure 4:**
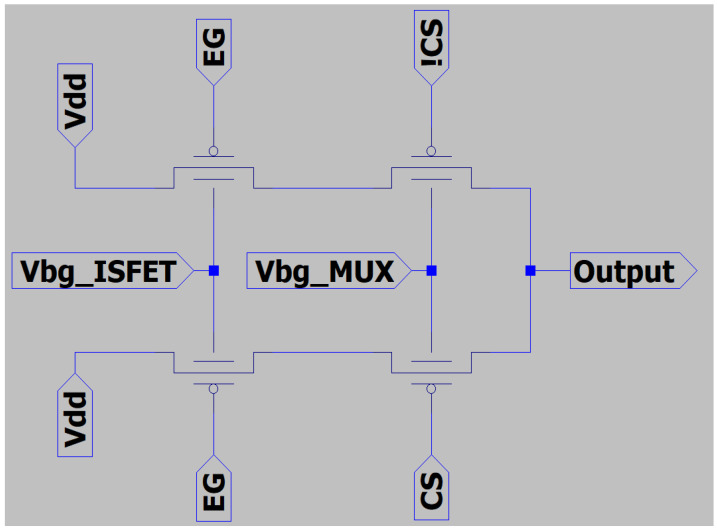
OTFT schematic of eight of our sensor chips. The chip contains eight similar structures implemented with different variants (transistor shape, dimension, etc.). Control signals (CS and !CS) are provided by external MCU.

Next, Figure 4 shows the schematic circuit implemented in our chips according to the architecture presented in Figure 2. All OTFTs are dual-gate but they will be implemented using different topologies, as shown in Table 1.

Our design strategy employs two different types of transistors for its further evaluation: interdigitated transistors and Corbino transistors, both of various sizes. The Corbino transistors were built in parallel to increase the equivalent gain (W/L) for this type of transistor (explained in more detail in the following paragraphs). The overall function of the circuit can be impacted by the performance characteristics that different transistor sizes may display. Hence, to make sure the circuit satisfies the specifications, the careful study and selection of the suitable transistor sizes are required. Increased gain, improved signal-to-noise ratio, and decreased overall transistor resistance are all made possible by the simultaneous employment of Corbino transistors. Also, the ability to parallel various numbers of Corbino transistors in various areas of the circuit provides designers with more choice when creating circuits with diverse requirements and standards.

**Figure 5:**
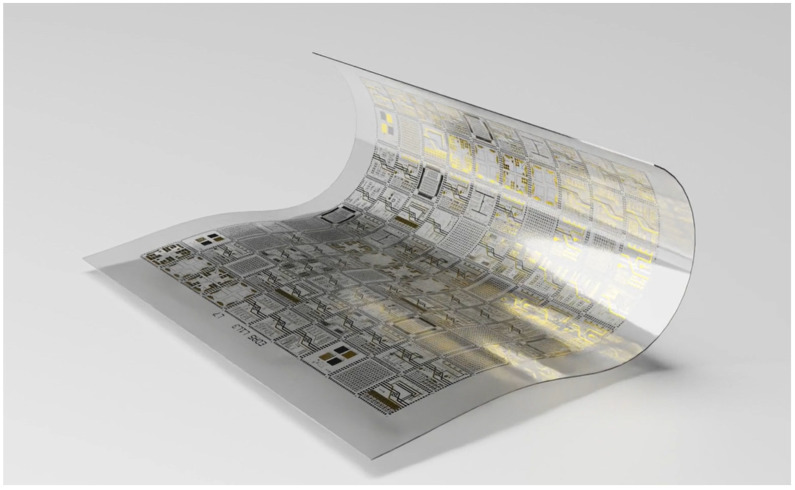
Implementation of several designs (including our sensors) on a flexible PEN substrate from SmartKem.

Figure 5 illustrates our design (together with other designs) implemented on a PEN (polyethylene naphthalate) substrate. The PEN substrate offers several advantages, such as flexibility, durability, and chemical resistance, making it suitable for various applications in flexible electronics. By utilizing the PEN substrate, the overall device becomes lightweight and bendable, allowing for seamless integration into wearable devices, flexible displays, and other portable electronic systems. The implementation of PEN demonstrates the feasibility of fabricating complex electronic circuits on flexible substrates, presenting new possibilities for the development of flexible and portable electronic devices.

**Figure 6:**
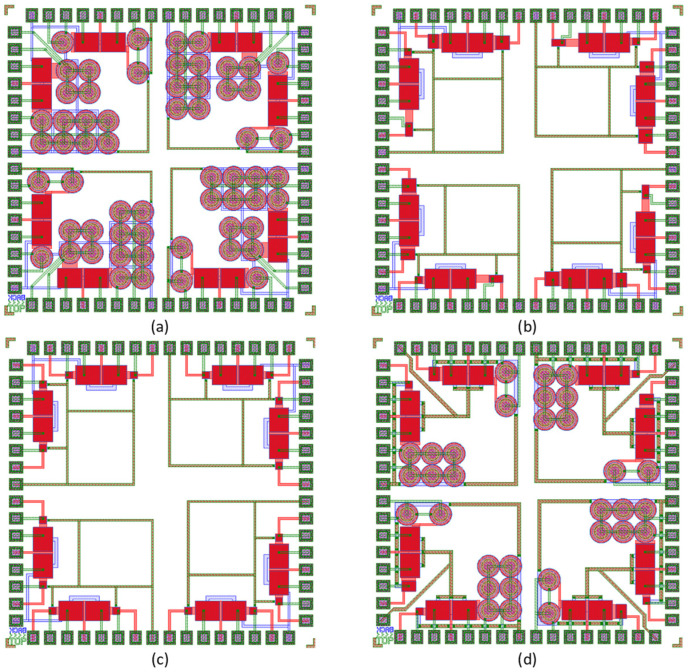
Four different designs with 16 ISFET sensors and different topologies: (**a**) Corbinos with varying W/L ratios, (**b**) interdigitated structures with different W/L ratios, (**c**) interdigitated structures with similar W/L ratios, and (**d**) a combination of Corbinos and interdigitated structures.

These dies were created with four primary ideas in mind. The first design is comprised of a die containing all Corbino transistors with changing W/L ratios, whereas the second design has all interdigitated transistors with varying W/L ratios. The third design uses identical W/L-sized interdigitated transistors. The fourth design combines Corbino and interdigitated transistors with comparable W/L ratios for both types. These designs are shown in Figure 6.

Corbino transistors (Figure 6a) have been used in parallel, with varying numbers of transistors paralleled in each section. Each Corbino transistor has a size of W = 1960 and L = 4. The first set has four parallel Corbino transistors, the second set has eight parallel Corbino transistors, the third set has two parallel Corbino transistors, and the last set has just one parallel Corbino transistor. 

In Figure 6b, the second design incorporates interdigitated transistors of varying sizes to produce varied gain (sensitivity) ranges on a single chip. The first circuit utilizes W/L ratios of 980, while the second circuit employs ratios of 1960, and the third circuit uses ratios of 3920. The final set of interdigitated transistors uses a W/L ratio of 2940. The interdigitated transistor design uses a variety of W/L ratios across all the transistors, in contrast to the Corbino transistor design, which uses parallel transistors. With this method, designers can alter the transistors’ gain and linearity in accordance with certain circuit needs, producing dependable and customized performance.

On the other hand, in Figure 6c, a different design approach is adopted using interdigitated transistors with a consistent W/L ratio of 980. This design simplifies the circuit design process by eliminating the complexity of varying transistor sizes. By using matching transistor sizes, a more uniform performance across the circuit is achieved. The redundancy offered by interdigitated transistors with the same W/L ratio becomes advantageous, as it enhances circuit reliability. In the event of a transistor failure, having identical-sized transistors allows for backup functionality, which is particularly critical in applications where a defective transistor can lead to severe consequences. However, it is important to note that this design strategy may limit the flexibility to customize the transistors’ performance properties. Therefore, a thorough optimization of the circuit characteristics is required to strike the right balance between benefits and drawbacks, as previously discussed. 

The advantages of Corbino and interdigitated transistors are combined (Figure 6d) in the interdigitated/Corbino architecture. Corbino transistors can be chosen in this architecture from a group of interdigitated transistors using MUXs. This provides a compromise between the advantages of both types of transistors and allows for greater flexibility in circuit design. The circuit’s flexibility to accommodate varied requirements and standards is further improved using interdigitated transistors with a variety of W/L ratios and the ability to parallel varying numbers of Corbino transistors in various regions of the circuit. The use of interdigitated transistors with the same W/L ratio also adds redundancy, enhancing the circuit’s overall reliability. Table 1 presents the various types and sizes of W/L (width-to-length) ratios employed in this design. As is evident from the data presented in Table 1, the Corbino ISFETs occupy significantly larger areas compared to the interdigitated ISFETs.

**Table 1:** Different types and sizes of W/L ratios in the different chips implemented.

Type	No. of Transistors	W/L	Area (µm^2^)	Chip
Corbino	1	1960/4 = 490	445,628	(a)
Corbino	2 parallel	3920/4 = 980	1,108,114	(a), (d)
Corbino	4 parallel	7840/4 = 1960	1,729,239	(a)
Corbino	6 parallel	11,760/4 = 2940	2,596,160	(d)
Corbino	8 parallel	15,680/4 = 3920	3,432,244	(a)
Interdigitated	1	3920/4 = 980	49,728	(b), (c)
Interdigitated	1	7840/4 = 1960	93,240	(b)
Interdigitated	1	11,760/4 = 2940	135,884	(b), (d)
Interdigitated	1	15,680/4 = 3920	177,240	(b)

**Figure 7:**
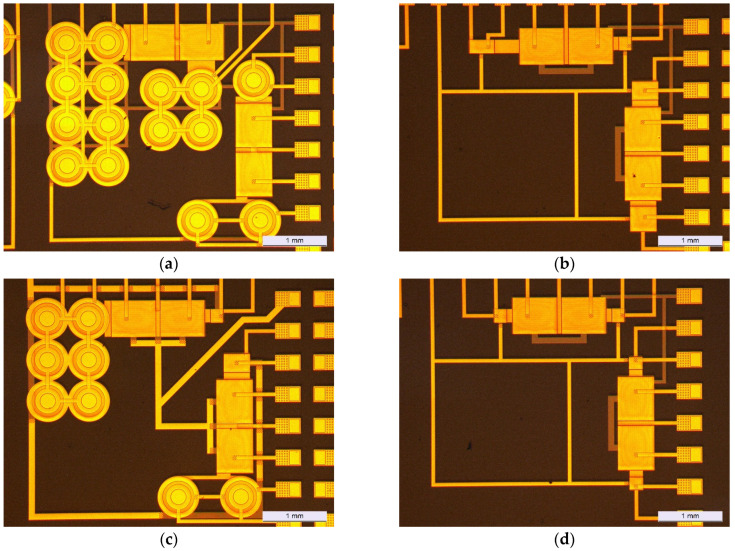
Images of parts of the dies fabricated by SmartKem: (**a**) all-Corbinos design (**b**) all-interdigitated with different W/L ratio design (**c**) interdigitated/Corbino design (**d**) all-interdigitated with same W/L ratio design.

Figure 7 illustrates the fabricated design for different dies, showcasing the variations and diversity in the physical realization of the design across multiple instances.

In order to simplify the characterization and test of the integrated circuit, the design includes four different equivalent structures placed at each of the quarters of the chip. A straightforward 90-degree rotation of the die on the packaging can provide access to equivalent transistors and circuits because each quarter of the dies is repeated four times on each die. Rotational access to similar transistors makes it possible to create intricate circuits that need many transistors with the same or similar properties and also provides fault-tolerance strategies related to the device failures already described.

## 7. OTFT Characterization 

To evaluate the performance of different transistors in the EG-ISFET sensor and OTFT MUX configuration, we conducted preliminary characterization experiments. These experiments provided vital insights into the behavior and efficiency of each transistor by extracting its V_th_. The experiments were conducted in tightly controlled environments with fixed bias, humidity, and temperature conditions. 

**Figure 8:**
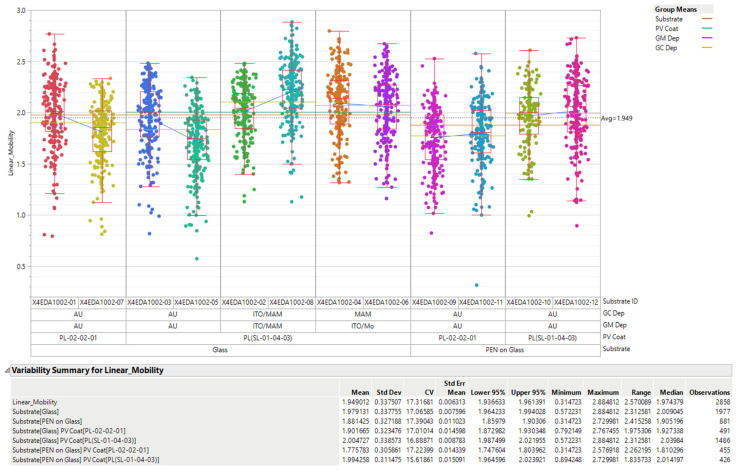
Mobility test results of dual-gate transistors on 12 distinct substrates.

Based on SmartKem technology, we were able to increase the mobility of OTFTs by applying the dual-bias approach on a dual gate. Using this technique, we obtained an increase in average OTFT mobility from 0.02 cm^2^ V^−1^s^−1^ [113] to 1.949 cm^2^ V^−1^s^−1^ with a variability of 18%. Figure 8 shows the mobility measurement for several transistors of the same run. The mobility value utilized in this test was computed based on the calculations provided by Brotherton [114].

These preliminary tests uncovered crucial information about the functionality and behavior of each transistor. During this test, the voltage of V_dd_ was maintained at a fixed value of −5 V. The V_bg_ and V_tg_ of the OTFTs were systematically varied to extract the V_th_ of each transistor. This information was instrumental in the further testing and adjustment of the sensor structure. As the back-gate voltage decreases, the threshold voltage increases. This behavior is evident in the data presented in Table 2, where the results for a back-gate voltage of −38 V are provided. According to the definition, Vth 1nA represents the gate voltage intercept when the drain current is equal to 1nA for a specific channel length (L) and width (W).

**Figure 9:**
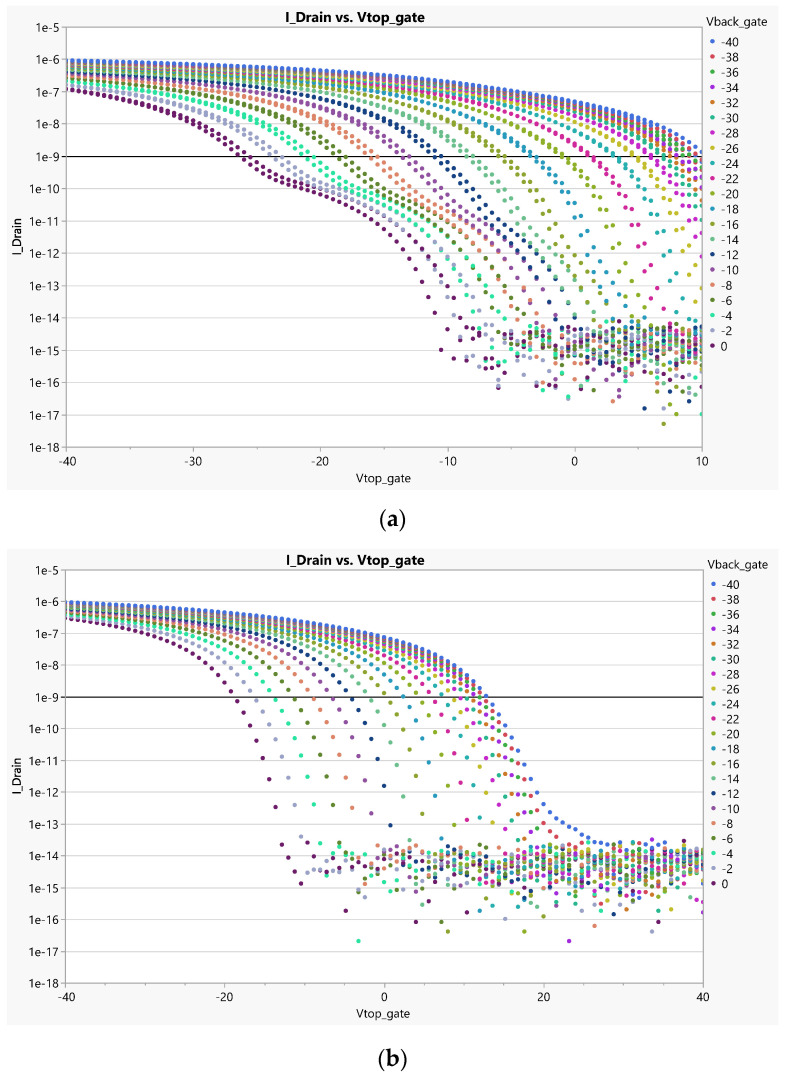
I–V curves for interdigitated OTFTs with (**a**) interdigitated with W/L = 3920/4 and (**b**) Corbino with W/L 1960 for different V_bg_ (between 0 V and −40 V). Each value of V_bg_ is represented by a different color on the graph.

In Figure 9a, the I-V curve illustrates that a drain current of 1nA intersects with the curve at a gate voltage of 9.5 V when the back-gate voltage is set to −38 V. This behavior is observed consistently across the other OTFT structures tested. However, an interesting observation can be made when the V_bg_ is increased to 0. In this case, it becomes evident that the interdigitated transistor with double the channel width (W) experiences significant stress and ultimately fails under the same voltage condition of −27 V. 

On the other hand, when examining the Corbino structure (Figure 9b), it demonstrates remarkable stability under different V_bg_ and V_tg_ conditions. The Corbino configuration, with its distinct design and electrode arrangement, exhibits a robust performance and maintains its functionality even under varying voltage biases.

**Table 2:** Variation in V_th_ for OTFTs at V_bg_ = −38 V.

Type	W/L	V_th,mean_ (V)
Corbino	490	12.8 ± 6%
Interdigitated	980	9.5 ± 5%
Interdigitated	1960	9.6 ± 5%
Interdigitated	2940	9.7 ± 5%
Interdigitated	3920	9.8 ± 5%
Interdigitated (MUX)	9800	9.9 ± 5%

Once we obtained the V_th_ values for each transistor in our EG-ISFET sensor and OTFT MUX configuration, we were able to determine the optimal effective voltages. To enhance the gain and precision of the sensor, we selected the most effective voltages for each transistor. Our careful selection of voltages ensured that the sensor could detect even the smallest changes in its environment. To ensure the sensor’s optimal performance in a variety of real-world applications, we employed these adjusted voltages in additional testing and calibration of the sensor.

The characterization of SmartKem transistors in OTFTs has provided valuable insights into their operational range, spanning from −5 V to +5 V, as shown in Table 2. By applying V_bg_ =−38 V to the of the MUXs, the top-gate voltage of −10 V and +8 V can be effectively utilized to control the switching of the MUXs for the ISFETs.

## 8. Circuit Tests

### 8.1. Testing Set-Up

To test the functionality of the MUX and EG-ISFET sensors, we used Analog Discovery 2 [115] and a standard power supply unit. We set up the measurement system such that V_DD_, V_SS_, and V_BG_ were fixed. Using the measurement system, we swept the voltage of the top gate of the EG-ISFET sensor and measured the corresponding output current, recording the values at each step of the sweep. We repeated the test for each sensor connected to the MUX, ensuring that all connections were maintained throughout the testing process. The tests were conducted under UV-light conditions, as it has enhanced the performance of OTFTs. 

### 8.2. Test Results

The sensor circuit functionality test was designed to verify the functionality of the EG-ISFET sensor and analog MUX. This involved ensuring that the MUX was correctly controlling the flow of signals coming from the different OTFT sensors and the output, and that the transfer function between each the OTFT MUX and the output current (voltage measured on a resistor load) was accurate. 

#### 8.2.1. Multiplexing Function

MUX OTFTs have been designed such that their W/L is much larger than that of the ISFET, to maximize sensitivity. Regarding our designs, all of them have the same-size W/L = 39,200/4 (all dimensions are in µm).

In order to test the multiplexing function, we set the voltages in the electrical nodes of Figure 4 according to Table 3.

**Figure 10:**
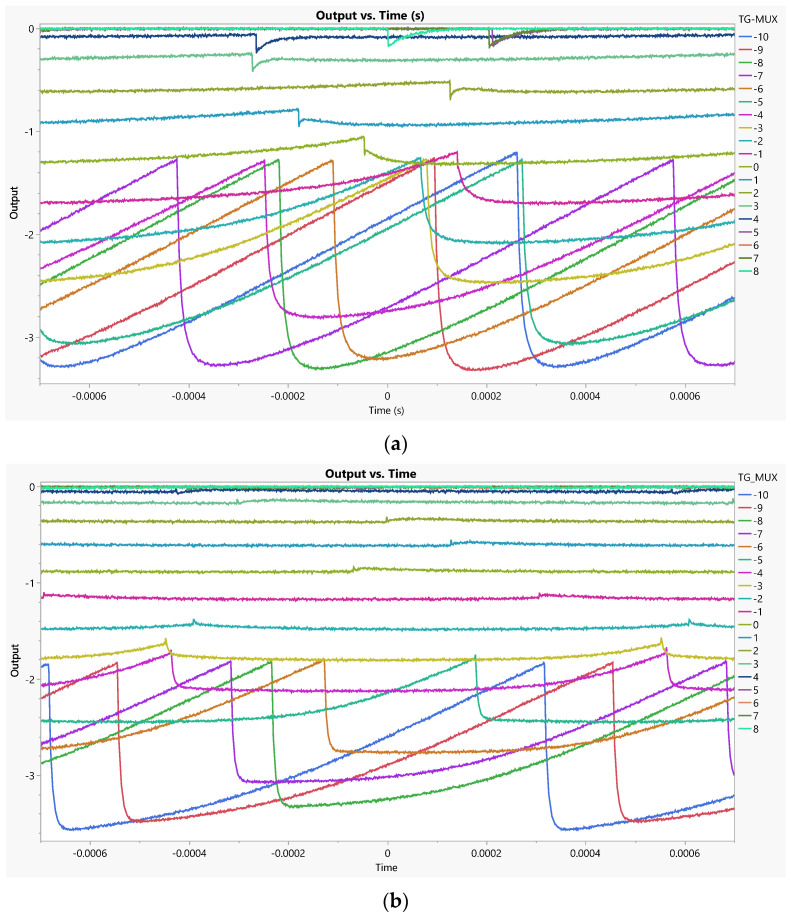
Signal propagation from the EG-ISFET gate to the output of the MUX for different top-gate voltages and (**a**) an interdigitated and (**b**) a Corbino topology (W/L = 3920/4 for both).

For testing purposes, we use a 1 MΩ resistor at the output to convert current to voltage. Figure 10 illustrates the application of several voltages to the top gate of the MUXs (equivalent to a switch selector), with each color representing a different voltage. The results show that the input signal (−5 V to +5 V ramp signal) applied to the EG-ISFET electrode goes through the MUX when its control voltage is −10 V (signifying an on state), giving the maximum output range (bottom blue curve). On the other hand, when the MUX voltage is +8 V (signaling an off state), it gives the minimum (0 ± 0.01 V) output range (top green curve), and therefore the MUX blocks the input signal and prevents it from getting to the output. In Figure 10, the input ramp signals (not shown) applied for the different MUX control voltages are not aligned, to better show the output signal behavior.

Corbino transistors show faster reactions from input to output; they are visible as less-curved responses. This is due to the larger output capacitance per width of interdigitated topologies.

#### 8.2.2. Global Sensing Function

In this section, we test the global transfer function from a voltage applied to the input of the ISFET (ideally coming from the external electrode) to the output current obtained after the analog MUX. 

**Figure 11:**
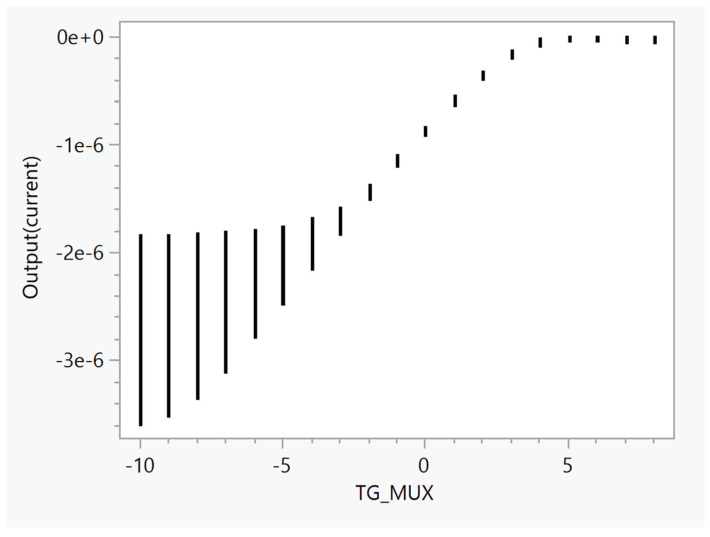
Global transfer function results showing the relationship between the input voltage applied to the ISFET and the corresponding output current after passing through the analog MUX.

For testing purposes, we use a 1 MΩ resistor at the output to convert the current to a voltage, to later obtain transconductance values for each of the circuit designs. The results of these tests are presented in Figure 11. Each bar within Figure 11 represents the output current obtained when the input signal is a ramp ranging from −5 V to 5 V.

#### 8.2.3. Transconductance Results

The transconductance findings from the experimental measurements are shown in the Table 4. 

Brotherton [114] suggested calculating the transconductance (*g_m_*) based on the following formula:

$$g_{m}\equiv \frac{dI_{d}}{dV_{G}}=\frac{{µ}_{n}WC_{i}V_{D}}{L}$$

By considering a capacitance value of *C_i_* = 6 nf/cm^2^, we can calculate the transconductance for different transistor designs. By calculating the transconductance, we can assess the effectiveness of the transistor in controlling the flow of current through the channel.

Table 4 shows the summary of these findings and offers a thorough comparison of the improvements made by various design configurations during the final test. The table makes it simple to evaluate each design’s performance and makes it easier to determine which design is the most profitable. It is possible to learn a lot about the amplification potential and effectiveness of the various configurations by analyzing the gains made by each design. For assessing and choosing the best design for a given application, the gain values achieved are crucial information. Measurements have been taken on eight different chips fabricated in the same run.

Measurements for both the eight Corbinos (5.76 × 10^−8^ ± 5%) and large (W/L = 15,680/4) interdigitated (8.90 × 10^−8^ ± 5%) designs exhibit a decrease in the output voltage, suggesting that the input voltage needs to be lower than −5 V to achieve the maximum output range and the corresponding accurate transconductance. 

Table 5 offers a comparison of the performance of the present design with results from three existing OTFT ISFETs found in the literature. The table encompasses mobility, transconductance, and area. In our method, utilizing a dual-gate approach, we were able to significantly increase the mobility. Our best case for transconductance values is slightly higher than the best of those found in the literature, while the area is in the same range as the others.

## 9. Conclusions

We have developed a novel sensor circuit structure by mixing extended ISFET sensors and multiplexers using dual-gate OTFTs, which presents a new model for implementing flexible and environment-friendly sensor chips. Multiplexing can be used to select different gains and sensitivities as well as to replace damaged devices (e.g., due to ESD failures). The ability to easily switch between ISFETs can be controlled by a simple MCU embedding ADC elementary analogs and digital blocks, thus improving the sensor system’s overall functionality.

The OTFT fabrication process is much simpler and cheaper than that of silicon-based technologies. Due to the lack of a CMOS process in the organic domain, dual-gate OTFTs are mandatory to build multiplexers that can block the signal coming from one of the ISFETs while processing the other one. The SmartKem 2.5 micron process allows for fabricating them. The use of digital patterning even removes the requirement of fabricating masks, what usually increases NRE costs. Still, some work must be conducted to obtain validated electrical models, to allow for better analysis and simulation. 

It is worth mentioning that OTFT ISFETs offer a recyclable advantage over conventional ISFETs, making them an environmentally friendly option for sensing applications. The use of organic materials for OTFT ISFETs in a low-temperature process enables the development of devices that can be easily recycled and disposed of in an eco-friendly manner. Unlike conventional ISFETs, which often contain rigid and non-recyclable materials like silicon substrates, OTFT ISFETs can be fabricated on flexible and recyclable substrates such as eco-friendly plastic or paper. This reduces the environmental impact associated with electronic waste and promotes a more sustainable approach to device manufacturing and deployment.

Our designs contain different Corbino and interdigitated structures, for which we have evaluated the output voltage, current, area, and transconductance under various input conditions and dimensions. The interdigitated transistors with a W/L ratio of 3920/4 stand out as the most advantageous in terms of transconductance among the tested configurations and have a 23% better performance than the Corbino topology with the same dimensions. 

Some further research could be conducted to tune the technology parameters (out of our capabilities), to optimize the operational ranges for voltage supply and the bias of the entire system.

## Figures and Tables

**Table 3:** Input values ranges for multiplexer test (dynamic).

I/O	Dir.	Unit	Value
V_DD_	Input	Voltage	−5
V_BG,mux_	Input	Voltage	−38
V_BG,sensor_	Input	Voltage	−38
V_TG,mux_	Input	Voltage	−10~+8
V_TG,sensor_	Input	Voltage	−5~+5
Source	Output	Current	Measure

**Table 4:** Transconductance comparison between different designs under V_TG, MUX_ = −10.

No.	Design Topology	g_m_
1	1 Corbino (W/L = 1960/4)	1.18 × 10^−7^ ± 5%
2	2 Corbinos (W/L = 3920/4)	1.21 × 10^−7^ ± 5%
3	4 Corbinos (W/L = 7840/4)	1.44 × 10^−7^ ± 5%
4	6 Corbinos (W/L = 11,760/4)	1.35 × 10^−7^ ± 5%
6	Interdigitated (W/L = 3920/4)	1.49 × 10^−7^ ± 5%
7	Interdigitated (W/L = 7840/4)	1.47 × 10^−7^ ± 5%
8	Interdigitated (W/L = 11,760/4)	1.09 × 10^−7^ ± 5%

**Table 5:** Transconductance comparison between different designs under V_TG, MUX_ = −10.

	Mobility (cm^2^V^−1^s^−1^)	Transconductance (S)	Area (mm^2^)
Proposed Method	1.949	1.49 × 10^−7^	0.4
Claramunt S., et al. [116]	0.55	1.4 × 10^−7^	-
Wu S.J., et al. [117]	0.33	0.99 × 10^−8^	0.5
Saini D., et al. [113]	0.02	0.3 × 10^−7^	0.2

## Data Availability

Data sharing not applicable.

## References

[1] Tixier-Mita A., Ihida S., Ségard B.-D., Cathcart G., Takahashi T., Fujita H., Toshiyoshi H. (2016). Review on thin-film transistor technology, its applications, and possible new applications to biological cells. Jpn. J. Appl. Phys..

[2] Kumar A., Goyal A.K., Gupta N. (2020). Review—Thin-Film Transistors (TFTs) for Highly Sensitive Biosensing Applications: A Review. ECS J. Solid State Sci. Technol..

[3] Weimer P.K. (1962). The TFT A New Thin-Film Transistor. Proc. IRE.

[4] Lechner B., Marlowe F., Nester E., Tults J. Liquid crystal matrix displays. Proceedings of the 1969 IEEE International Solid-State Circuits Conference.

[5] Le Comber P., Spear W., Ghaith A. (1979). Amorphous-silicon field-effect device and possible application. Electron. Lett..

[6] Tiedje T., Abeles B., Morel D.L., Moustakas T.D., Wronski C.R. (1980). Electron drift mobility in hydrogenated a-Si. Appl. Phys. Lett..

[7] Depp S., Juliana A., Huth B. Polysilicon FET devices for large area input/output applications. Proceedings of the 1980 International Electron Devices Meeting.

[8] Gao X., Lin L., Liu Y., Huang X. (2015). LTPS TFT Process on Polyimide Substrate for Flexible AMOLED. J. Disp. Technol..

[9] Serikawa T., Shirai S., Okamoto A., Suyama S. (1989). Low temperature fabrication of high mobility poly-Si TFTs for large area LCDs. IEEE Trans. Electron Devices.

[10] Miyata Y., Furuta M., Yoshioka T.Y.T., Kawamura T.K.T. (1992). Low-Temperature Polycrystalline Silicon Thin-Film Transistors for Large-Area Liquid Crystal Display. Jpn. J. Appl. Phys..

[11] Biggs J., Myers J., Kufel J., Ozer E., Craske S., Sou A., Ramsdale C., Williamson K., Price R., White S. (2021). A natively flexible 32-bit Arm microprocessor. Nature.

[12] Ebisawa F., Kurokawa T., Nara S. (1983). Electrical properties of polyacetylene/polysiloxane interface. J. Appl. Phys..

[13] Drury C.J., Mutsaers C.M.J., Hart C.M., Matters M., de Leeuw D.M. (1998). Low-cost all-polymer integrated circuits. Appl. Phys. Lett..

[14] Guo X., Xu Y., Ogier S., Ng T.N., Caironi M., Perinot A., Li L., Zhao J., Tang W., Sporea R.A. (2017). Current Status and Opportunities of Organic Thin-Film Transistor Technologies. IEEE Trans. Electron Devices.

[15] Jenkins E. (2017). High-performance apparel for outdoor pursuits. High-Performance Apparel: Materials, Development, and Applications.

[16] Koezuka H., Tsumura A., Ando T. (1987). Field-effect transistor with polythiophene thin film. Synth. Met..

[17] Park C.B., Kim K.M., Lee J.E., Na H., Yoo S.S., Yang M.S. (2014). Flexible electrophoretic display driven by solution-processed organic TFT with highly stable bending feature. Org. Electron..

[18] Chen C.-M., Anastasova S., Zhang K., Gil Rosa B., Lo B.P.L., Assender H.E., Yang G.-Z. (2019). Towards Wearable and Flexible Sensors and Circuits Integration for Stress Monitoring. IEEE J. Biomed. Heal. Inform..

[19] Myny K., Steudel S., Smout S., Vicca P., Furthner F., van der Putten B., Tripathi A., Gelinck G., Genoe J., Dehaene W. (2010). Organic RFID transponder chip with data rate compatible with electronic product coding. Org. Electron..

[20] Fiore V., Battiato P., Abdinia S., Jacobs S., Chartier I., Coppard R., Klink G., Cantatore E., Ragonese E., Palmisano G. (2015). An Integrated 13.56-MHz RFID Tag in a Printed Organic Complementary TFT Technology on Flexible Substrate. IEEE Trans. Circuits Syst. I Regul. Pap..

[21] Shiwaku R., Matsui H., Nagamine K., Uematsu M., Mano T., Maruyama Y., Nomura A., Tsuchiya K., Hayasaka K., Takeda Y. (2018). A Printed Organic Amplification System for Wearable Potentiometric Electrochemical Sensors. Sci. Rep..

[22] Minemawari H., Yamada T., Matsui H., Tsutsumi J., Haas S., Chiba R., Kumai R., Hasegawa T. (2011). Inkjet printing of single-crystal films. Nature.

[23] Venkateshvaran D., Nikolka M., Sadhanala A., Lemaur V., Zelazny M., Kepa M., Hurhangee M., Kronemeijer A.J., Pecunia V., Nasrallah I. (2014). Approaching disorder-free transport in high-mobility conjugated polymers. Nature.

[24] Sirringhaus H. (2014). 25th Anniversary Article: Organic Field-Effect Transistors: The Path Beyond Amorphous Silicon. Adv. Mater..

[25] Shao Y., Xiao Z., Bi C., Yuan Y., Huang J. (2014). Origin and elimination of photocurrent hysteresis by fullerene passivation in CH3NH3PbI3 planar heterojunction solar cells. Nat. Commun..

[26] Fan H., Zou S., Gao J., Chen R., Ma Q., Ma W., Zhang H., Chen G., Huo X., Liu Z. (2020). High-mobility organic single-crystalline transistors with anisotropic transport based on high symmetrical “H”-shaped heteroarene derivatives. J. Mater. Chem. C.

[27] Paterson A.F., Singh S., Fallon K.J., Hodsden T., Han Y., Schroeder B.C., Bronstein H., Heeney M., McCulloch I., Anthopoulos T.D. (2018). Recent Progress in High-Mobility Organic Transistors: A Reality Check. Adv. Mater..

[28] Kim N.S., Austin T., Blaauw D., Mudge T., Flautner K., Hu J.S., Irwin M., Kandemir M., Narayanan V. (2003). Leakage current: Moore’s law meets static power. Computer.

[29] Steudel S., Myny K., De Vusser S., Genoe J., Heremans P. (2006). Patterning of organic thin film transistors by oxygen plasma etch. Appl. Phys. Lett..

[30] Tang W., Zhao J., Feng L., Yu P., Zhang W., Guo X. (2014). Top-Gate Dry-Etching Patterned Polymer Thin-Film Transistors With a Protective Layer on Top of the Channel. IEEE Electron Device Lett..

[31] Borchert J.W., Zschieschang U., Letzkus F., Giorgio M., Weitz R.T., Caironi M., Burghartz J.N., Ludwigs S., Klauk H. (2020). Flexible low-voltage high-frequency organic thin-film transistors. Sci. Adv..

[32] Chang T.K., Lin C.W., Chang S. (2019). LTPO TFT technology for amoleds. Dig. Tech. Pap..

[33] Sameshima T., Sekiya M., Usui S. (1986). XeCl Excimer Laser Annealing used in the Fabrication of Poly-Si TFTs. IEEE Electron Device Lett..

[34] Klauk H., Halik M., Zschieschang U., Schmid G., Radlik W., Weber W. (2002). High-mobility polymer gate dielectric pentacene thin film transistors. J. Appl. Phys..

[35] Forrest S.R. (2004). The path to ubiquitous and low-cost organic electronic appliances on plastic. Nature.

[36] Zhang Y., Li D., Jiang C. (2013). Influence of grain size at first monolayer on bias-stress effect in pentacene-based thin film transistors. Appl. Phys. Lett..

[37] Huang S.-P., Chen H.-C., Chen P.-H., Zheng Y.-Z., Chu A.-K., Shih Y.-S., Wang Y.-X., Wu C.-C., Chen Y.-A., Sun P.-J. (2020). Effect of ELA Energy Density on Self-Heating Stress in Low-Temperature Polycrystalline Silicon Thin-Film Transistors. IEEE Trans. Electron Devices.

[38] Zheng Y.-Z., Huang S.-P., Chen P.-H., Chang T.-C., Tsai T.-M., Chu A.-K., Hung Y.-H., Shih Y.-S., Wang Y.-X., Wu C.-C. (2020). Enhancement of Mechanical Bending Stress Endurance Using an Organic Trench Structure in Foldable Polycrystalline Silicon TFTs. IEEE Electron Device Lett..

[39] Lee J.-H., Kim D.-H., Yang D.-J., Hong S.-Y., Yoon K.-S., Hong P.-S., Jeong C.-O., Park H.-S., Kim S.Y., Lim S.K. (2008). 42.2: World’s Largest (15-inch) XGA AMLCD Panel Using IGZO Oxide TFT. Dig. Tech. Pap..

[40] Kim M., Ryu S.U., Park S.A., Choi K., Kim T., Chung D., Park T. (2020). Donor–Acceptor-Conjugated Polymer for High-Performance Organic Field-Effect Transistors: A Progress Report. Adv. Funct. Mater..

[41] Fratini S., Nikolka M., Salleo A., Schweicher G., Sirringhaus H. (2020). Charge transport in high-mobility conjugated polymers and molecular semiconductors. Nat. Mater..

[42] Phan H., Wang M., Bazan G.C., Nguyen T.-Q. (2015). Electrical Instability Induced by Electron Trapping in Low-Bandgap Donor-Acceptor Polymer Field-Effect Transistors. Adv. Mater..

[43] Wu X., Jia R., Jie J., Zhang M., Pan J., Zhang X., Zhang X. (2019). Air Effect on the Ideality of p-Type Organic Field-Effect Transistors: A Double-Edged Sword. Adv. Funct. Mater..

[44] Yang Z., Guo C., Shi C., Wang D.K., Zhang T., Zhu Q., Lu Z.H. (2020). Improving Bias-Stress Stability of p-Type Organic Field-Effect Tran-sistors by Constructing an Electron Injection Barrier at the Drain Electrode/Semiconductor Interfaces. ACS Appl. Mater. Interfaces.

[45] Nikolka M., Nasrallah I., Rose B., Ravva M.K., Broch K., Sadhanala A., Harkin D., Charmet J., Hurhangee M., Brown A. (2017). High operational and environmental stability of high-mobility conjugated polymer field-effect transistors through the use of molecular additives. Nat. Mater..

[46] Phan H., Ford M.J., Lill A.T., Wang M., Bazan G.C., Nguyen T.-Q. (2017). Improving Electrical Stability and Ideality in Organic Field-Effect Transistors by the Addition of Fullerenes: Understanding the Working Mechanism. Adv. Funct. Mater..

[47] Xu Y., Sun H., Shin E.-Y., Lin Y.-F., Li W., Noh Y.-Y. (2016). Planar-Processed Polymer Transistors. Adv. Mater..

[48] Wu X., Jia R., Pan J., Wang J., Deng W., Xiao P., Zhang X., Jie J. (2021). Improving Ideality of P-Type Organic Field-Effect Transistors via Preventing Undesired Minority Carrier Injection. Adv. Funct. Mater..

[49] Wang C., Dong H., Jiang L., Hu W. (2018). Organic semiconductor crystals. Chem. Soc. Rev..

[50] Liu C., Jang J., Xu Y., Kim H.-J., Khim D., Park W.-T., Noh Y.-Y., Kim J.-J. (2014). Effect of Doping Concentration on Microstructure of Conjugated Polymers and Characteristics in N-Type Polymer Field-Effect Transistors. Adv. Funct. Mater..

[51] Lüssem B., Keum C.M., Kasemann D., Naab B., Bao Z., Leo K. (2016). Doped Organic Transistors. Chem. Reviews. Am. Chem. Soc..

[52] Bausells J., Carrabina J., Errachid A., Merlos A. (1999). Ion-sensitive field-effect transistors fabricated in a commercial CMOS technology. Sens. Actuators B Chem..

[53] Hansen C.L., Skordalakest E., Berger J.M., Quake S.R. (2002). A robust and scalable microfluidic metering method that allows protein crystal growth by free interface diffusion. Proc. Natl. Acad. Sci. USA.

[54] Takayama S., Ostuni E., LeDuc P., Naruse K., Ingber D.E., Whitesides G.M. (2001). Subcellular positioning of small molecules. Nature.

[55] Son J., Samuel R., Gale B.K., Carrell D.T., Hotaling J.M. (2017). Separation of sperm cells from samples containing high concentrations of white blood cells using a spiral channel. Biomicrofluidics.

[56] Jafek A.R., Harbertson S., Brady H., Samuel R., Gale B.K. (2018). Instrumentation for xPCR Incorporating qPCR and HRMA. Anal. Chem..

[57] Bange A., Halsall H.B., Heineman W.R. (2005). Microfluidic immunosensor systems. Biosens. Bioelectron..

[58] Guo M.T., Rotem A., Heyman J.A., Weitz D.A. (2012). Droplet microfluidics for high-throughput biological assays. Lab Chip.

[59] Safdar M., Jänis J., Sánchez S. (2016). Microfluidic fuel cells for energy generation. Lab Chip.

[60] Xia Y., Whitesides G.M. (1998). Soft lithography. Annu. Rev. Mater. Sci..

[61] McDonald J.C., Duffy D.C., Anderson J.R., Chiu D.T., Wu H., Schueller O.J., Whitesides G.M. (2000). Fabrication of microfluidic systems in poly(dimethylsiloxane). Electrophoresis.

[62] Qin D., Xia Y., Whitesides G.M. (2010). Soft lithography for micro- and nanoscale patterning. Nat. Protoc..

[63] Faustino V., Catarino S.O., Lima R., Minas G. (2016). Biomedical microfluidic devices by using low-cost fabrication techniques: A review. J. Biomech..

[64] Telting-Diaz M., Bakker E. (2002). Mass-Produced Ionophore-Based Fluorescent Microspheres for Trace Level Determination of Lead Ions. Anal. Chem..

[65] Wang X., Zhang X. (2013). Electrochemical co-reduction synthesis of graphene/nano-gold composites and its application to electro-chemical glucose biosensor. Electrochim Acta..

[66] Privett B.J., Shin J.H., Schoenfisch M.H. (2010). Electrochemical sensors. Anal Chem..

[67] Chen C., Kotyk J.F.K., Sheehan S.W. (2018). Progress toward Commercial Application of Electrochemical Carbon Dioxide Reduction. Chem.

[68] Wang Y., Xu H., Zhang J., Li G. (2008). Electrochemical Sensors for Clinic Analysis. Sensors.

[69] Nurul Islam M., Mazhari B. (2013). Organic thin film transistors with asymmetrically placed source and drain contact. Org. Electron..

[70] Jeon J.H., Cho W.J. (2020). High-performance extended-gate ion-sensitive field-effect transistors with multi-gate structure for trans-parent, flexible, and wearable biosensors. Sci. Technol. Adv. Mater..

[71] Zeng R., Zhang J., Sun C., Xu M., Zhang S.-L., Wu D. (2018). A reference-less semiconductor ion sensor. Sens. Actuators B Chem..

[72] Vonau W., Gerlach F., Herrmann S. (2010). Conception of a new technique in cell cultivation using a lab-on-chip aided miniaturised device with calibratable electrochemical sensors. Microchim. Acta.

[73] Parizi K.B., Xu X., Pal A., Hu X., Wong H.S.P. (2017). ISFET pH Sensitivity: Counter-Ions Play a Key Role. Sci. Rep..

[74] Bouftas M. (2021). A Systematic Review on the Feasibility of Salivary Biomarkers for Alzheimer’s Disease. J. Prev. Alzheimer’s Dis..

[75] Lau H.-C., Lee I.-K., Ko P.-W., Lee H.-W., Huh J.-S., Cho W.-J., Lim J.-O. (2015). Non-Invasive Screening for Alzheimer’s Disease by Sensing Salivary Sugar Using Drosophila Cells Expressing Gustatory Receptor (Gr5a) Immobilized on an Extended Gate Ion-Sensitive Field-Effect Transistor (EG-ISFET) Biosensor. PLoS ONE.

[76] McKinley B.A. (2008). ISFET and Fiber Optic Sensor Technologies: In Vivo Experience for Critical Care Monitoring. Chem. Rev..

[77] Baghini M.S., Vilouras A., Douthwaite M., Georgiou P., Dahiya R. (2022). Ultra-thin ISFET-based sensing systems. Electrochem. Sci. Adv..

[78] Pirson T., Delhaye T., Pip A., Le Brun G., Raskin J.-P., Bol D. The Environmental Footprint of IC Production: Meta-Analysis and Historical Trends. Proceedings of the ESSDERC 2022-IEEE 52nd European Solid-State Device Research Conference (ESSDERC).

[79] Ogier S., Sharkey D., Carreras A., Tsai S. (2022). 69-3: Opportunities for High-Performance Display Manufacturing Enabled by OTFTs Using an 80*C Maximum Process Temperature. SID Symp. Dig. Tech. Pap..

[80] Zheng Y.-Z., Chen Y.-A., Chen P.-H., Chang T.-C., Hung Y.-H., Zhou K.-J., Tu Y.-F., Wang Y.-X., Chen J.-J., Wu C.-C. (2022). Physical Mechanism of the Mechanical Bending of High-Performance Organic TFTs and the Effect of Atmospheric Factors. ACS Appl. Electron. Mater..

[81] Zschieschang U., Klauk H., Borchert J.W. (2023). High-Resolution Lithography for High-Frequency Organic Thin-Film Transistors. Adv. Mater. Technol..

[82] Cavallari M.R., Pastrana L.M., Sosa C.D.F., Marquina A.M.R., Izquierdo J.E.E., Fonseca F.J., de Amorim C.A., Paterno L.G., Kymissis I. (2020). Organic Thin-Film Transistors as Gas Sensors: A Review. Materials.

[83] Bilgaiyan A., Cho S.-I., Abiko M., Watanabe K., Mizukami M. (2021). Flexible, high mobility short-channel organic thin film transistors and logic circuits based on 4H–21DNTT. Sci. Rep..

[84] Gupta S., Singh M.K. (2021). Key aspects affecting the performances of high-K dielectrics based single-gate and dual-gate OTFTs. Mater. Today Proc..

[85] Kumar B., Kaushik B.K., Negi Y.S., Goswami V. (2014). Single and dual gate OTFT based robust organic digital design. Microelectron. Reliab..

[86] Ha T.-J., Kiriya D., Chen K., Javey A. (2014). Highly Stable Hysteresis-Free Carbon Nanotube Thin-Film Transistors by Fluorocarbon Polymer Encapsulation. ACS Appl. Mater. Interfaces.

[87] Dou W., Tan Y. (2020). Dual-gate low-voltage transparent electric-double-layer thin-film transistors with a top gate for threshold voltage modulation. RSC Adv..

[88] Duvvury C., Amerasekera A. (1993). ESD: A pervasive reliability concern for IC technologies. Proc. IEEE.

[89] Kleinman D.A., Schawlow A.L. (1960). Corbino disk. J. Appl. Phys..

[90] Guzenko V.A., Akabori M., Schäpers T., Cabañas S., Sato T., Suzuki T., Yamada S. (2006). Weak antilocalization measurements on a 2-dimensional electron gas in an InGaSb/InAlSb heterostructure. Phys. Status Solidi.

[91] Klauk H., Gundlach D., Nichols J., Jackson T. (1999). Pentacene organic thin-film transistors for circuit and display applications. IEEE Trans. Electron. Devices.

[92] Arnal A., Martínez-Domingo C., Ogier S., Terés L., Ramon E. (2019). Monotype Organic Dual Threshold Voltage Using Different OTFT Geometries. Crystals.

[93] Byun Y.H., Den Boer W., Yang M., Gu T. (1996). An amorphous silicon TFT with annu-lar-shaped channel and reduced gate-source capacitance. IEEE Trans. Electron. Devices.

[94] Munteanu D., Cristoloveanu S., Hovel H. (1999). Circular pseudo-metal oxide semiconductor field effect transistor in sili-con-on-insulator analytical model, simulation, and measurements. Electrochem. Solid-State Lett..

[95] Zhao C., Fung T.C., Kanicki J. (2016). Half-Corbino short-channel amorphous In–Ga–Zn–O thin-film transistors with a-SiOx or a-SiOx/a-SiNx passivation layers. Solid State Electron..

[96] Kong J., Liu C., Li X., Ou H., She J., Deng S., Chen J. (2023). Characteristics of Offset Corbino Thin Film Transistor: A Physical Model. Electronics.

[97] Mativenga M., Geng D., Um J.K., Mruthyunjaya R.K., Heiler G.N., Tredwell T.J., Jang J. (2014). 49.2: Corbino TFTs for Large-Area AMOLED Displays. SID Symp. Dig. Tech. Pap..

[98] Das M.B., Josephy R.D. (1971). High Frequency, High Power Igfet with Interdigital Electrodes and Plural Looped Gate. U.S. Patent.

[99] Tisserant J.-N., Wicht G., Göbel O.F., Bocek E., Bona G.-L., Geiger T., Hany R., Mezzenga R., Partel S., Schmid P. (2013). Growth and Alignment of Thin Film Organic Single Crystals from Dewetting Patterns. ACS Nano.

[100] Bi S., He Z., Chen J., Li D. (2015). Solution-grown small-molecule organic semiconductor with enhanced crystal alignment and areal coverage for organic thin film transistors. AIP Adv..

[101] Fujisaki Y., Takahashi D., Nakajima Y., Nakata M., Tsuji H., Yamamoto T. (2015). Alignment Control of Patterned Organic Semicon-ductor Crystals in Short-Channel Transistor Using Unidirectional Solvent Evaporation Process. IEEE Trans. Electron. Devices.

[102] Genco E., Fattori M., Harpe P.J.A., Modena F., Viola F.A., Caironi M., Cantatore E. (2022). A 4 × 4 Biosensor Array With a 42- μ W/Channel Mul-tiplexed Current Sensitive Front-End Featuring 137-dB DR and Zeptomolar Sensitivity. IEEE Open J. Solid-State Circuits Soc..

[103] Zeng J., Kuang L., Miscourides N., Georgiou P. (2020). A 128 × 128 Current-Mode Ultra-High Frame Rate ISFET Array with In-Pixel Calibration for Real-Time Ion Imaging. IEEE Trans. Biomed. Circuits Syst..

[104] Zeng J., Miscourides N., Georgiou P. A 128 × 128 Current-Mode Ultra-High Frame Rate ISFET Array for Ion Imaging. Proceedings of the 2018 IEEE International Symposium on Circuits and Systems (ISCAS).

[105] Cong Y., Xu M., Zhao D., Wu D. A 3600 × 3600 large-scale ISFET sensor array for high-throughput pH sensing. Proceedings of the 2017 IEEE 12th International Conference on ASIC (ASICON).

[106] Chan W.P., Premanode B., Toumazou C. (2009). 64 pH-ISFET averaging array employing global negative current feedback. Electron. Lett..

[107] Eversmann B., Jenkner M., Hofmann F., Paulus C., Brederlow R., Holzapfl B. (2003). A 128 128 CMOS Biosensor Array for Extra-cellular Recording of Neural Activity. IEEE J. Solid-State Circuits.

[108] Bao B., Karnaushenko D.D., Schmidt O.G., Song Y., Karnaushenko D. (2022). Active Matrix Flexible Sensory Systems: Materials, Design, Fabrication, and Integration. Adv. Intell. Syst..

[109] Fattori M., Cardarelli S., Fijn J., Harpe P., Charbonneau M., Locatelli D., Lombard S., Laugier C., Tournon L., Jacob S. (2022). A printed proximity-sensing surface based on organic pyroelectric sensors and organic thin-film transistor electronics. Nat. Electron..

[110] Luo Z., Peng B., Zeng J., Yu Z., Zhao Y., Xie J., Lan R., Ma Z., Pan L., Cao K. (2021). Sub-thermionic, ultra-high-gain organic transistors and circuits. Nat. Commun..

[111] Lafaye C., Rovira M., Demuru S., Wang S., Kim J., Kunnel B.P., Besson C., Fernandez-Sanchez C., Serra-Graells F., Margarit-Taulé J.M. Real-time smart multisensing wearable platform for monitoring sweat biomarkers during exercise. Proceedings of the BioCAS 2022—IEEE Biomedical Circuits and Systems Conference: Intelligent Biomedical Systems for a Better Future.

[112] Cisneros-Fernandez J., Guimera-Brunet A., Garcia-Cortadella R., Schafer N., Garrido J.A., Teres L., Serra-Graells F. A 1024-Channel GFET 10-bit 5-kHz 36-µW Read-Out Integrated Circuit for Brain JLECoG. Proceedings of the ICECS 2020—27th IEEE International Conference on Electronics, Circuits and Systems.

[113] Saini D., Saini S., Negi S. Modelling and comparison of single gate and dual gate organic thin film transistor. Proceedings of the 2016 International Conference on Emerging Trends in Communication Technologies, ETCT.

[114] Brotherton S.D. (2013). Introduction to thin film transistors: Physics and technology of TFTs. Introduction to Thin Film Transistors: Physics and Technology of TFTs.

[115] Digilent Digilent. Analog Discovery 2—Digilent Reference Manual. https://digilent.com/reference/test-and-measurement/analog-discovery-2/reference-manual.

[116] Claramunt S., Palau G., Arnal A., Crespo-Yepes A., Porti M., Ogier S. (2023). Exploitation of OTFTs variability for PUFs imple-mentation and impact of aging. Solid State Electron..

[117] Wu S.-J., Wu Y.-C., Tsai H.-H., Liao H.-H., Juang Y.-Z., Lin C.-H., Shang-Jing W., Yung-Chen W., Hann-Huei T., Hsin-Hao L. ISFET-based pH sensor composed of a high transconductance CMOS chip and a disposable touch panel film as the sensing layer. Proceedings of the 2015 IEEE SENSORS.

